# COVID-19: Why Algeria’s Case Fatality Rate Seems to be Among the Highest in the World?

**DOI:** 10.5152/eurasianjmed.2021.20192

**Published:** 2021-06

**Authors:** Mohamed Lounis

**Affiliations:** Department of Agro-veterinary Science, Faculty of Natural and Life Sciences, University of Ziane Achour, BP 3117, Road of Moudjbara, Djelfa 17000, Algeria

Dear Editor:

The spread of the novel Coronavirus disease (COVID-19) has not slowed down or stopped across the world since it started in Wuhan (China) on December 2019. With more than 8 million positive cases in almost all countries and regions and more than 480,000 deaths, the disease has exceeded all expectations.^1^

From the first COVID-19 positive case reported on February 25, Algeria accounts 11,268 cases (June 16) at present. This number makes it the fifth most affected country in Africa after South Africa, Egypt, Nigeria, and Ghana.^1^,^2^ However, it is ranked third in the number of deaths, with 799 cases translating to a fatality rate of 7.1 %, which is the 13^th^ highest in the world after those reported in the most affected countries such as France (15.5%), United Kingdom (14.1%), Italy (14.5 %), and Spain (11.1 %).^3^ Furthermore, from April 12 to April 15, Algeria has shocked the world by reporting the highest fatality rate (number of deaths/number of affected cases) in the world (between 15.1% and 15. 8%).^1^

The question is: Has Algeria really recorded the highest fatality rate in the world? In this manuscript, we will try to explain why Algeria recorded such a high fatality rate.

First, Algeria has a relatively weak health care system, with a lack of adequate diagnostic facilities and hospitals with no proper resources to handle infectious diseases. Therefore, in 2019, the Global Health Security index identified Algeria as one of the countries that are not fully prepared to respond to health crisis of global importance.^4^ However, in a recent study, Algeria, along with South Africa and Egypt, was identified as one with the highest capacity to respond to outbreaks in Africa.^5^

Second, in the initial days after the apparition of the disease, only the Institut Pasteur d’Algérie (Algiers) was approved to conduct tests. The number tests performed does not exceed 60 per day. After other laboratories were renovated and adapted in different departments, the number of tests performed reached approximately 400 tests per day, with an average of 200 tests per day since the first reported case. Until now, approximately 30,000 tests have been conducted. However, the number of positive cases seems to be higher than the declared cases. This improvement in the screening capacities has been associated with a decrease in the fatality rate (Figure 1). This number is still extremely low when compared with the most affected African countries like South Africa (1,200,736), Ghana (258,010), Nigeria (103,799), and Egypt (135,000).^1^

Third, the number of suspected cases diagnosed with computed tomography scan and treated is estimated to be 14,005, increasing the number of positive cases to 23,532, which in turn decreases the estimated fatality rate to 3.4 %. This is lower than the average global fatality rate (5.4%).^2^

Finally, this rate varies significantly among different departments, and the 10 most affected departments (more than 200 cases) have shown a fatality rate ranging from 2% to 15.1%.^2^

All the above explanations indicate that the high fatality rate recorded in Algeria is to be treated with some caution awaiting the final ranking.

## Figures and Tables

**Figure 1:**
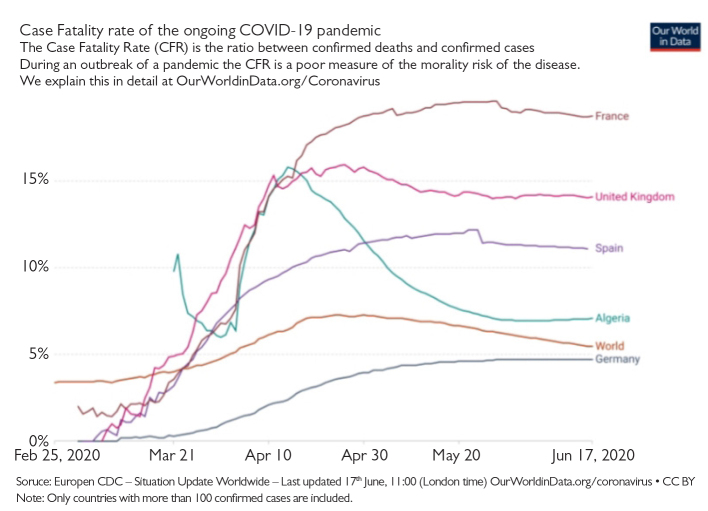
Fatality rates of the ongoing COVID-19 pandemic in Algeria and other countries Soruce: Europen CDC – Situation Update Worldwide – Last updated 17^th^ June, 11:00 (London time) OurWorldinData.org/coronavirus • CC BY Note: Only countries with more than 100 confirmed cases are included.
